# Magnesium Intake Predicts Bone Turnover in Postmenopausal Black South African Women

**DOI:** 10.3390/nu11102519

**Published:** 2019-10-18

**Authors:** Hattie H. Wright, Marlena C. Kruger, Willem D. Schutte, Edelweiss Wentzel-Viljoen, Iolanthe M. Kruger, Herculina S. Kruger

**Affiliations:** 1 School of Health and Sport Sciences, University of the Sunshine Coast, Maroochydore 4556, Australia; 2 Centre of Excellence for Nutrition, North-West University, Potchefstroom 2520, South Africa; edelweiss.wentzel-viljoen@nwu.ac.za (E.W.-V.); Salome.Kruger@nwu.ac.za (H.S.K.); 3 School of Health Sciences, Massey University, Palmerston North 4410, New Zealand; M.C.Kruger@massey.ac.nz; 4 Centre for Business Mathematics and Informatics, North-West University, Potchefstroom 2520, South Africa; Wd.Schutte@nwu.ac.za; 5 Africa Unit for Transdisciplinary Health Research, North-West University, Potchefstroom 2520, South Africa; Lanthe.Kruger@nwu.ac.za

**Keywords:** dietary patterns, bone mineral density, bone resorption, post-menopausal, Black women, dietary calcium, urban

## Abstract

This prospective study investigated the association between nutrient intake, dietary patterns, and changes in bone turnover and bone mineral density (BMD) in postmenopausal urban black South African women over two years. These women (*n* = 144) underwent BMD measurements at the distal radius, lumbar spine, femoral neck (FN), as well as a biochemical analysis which included the parathyroid hormone (PTH), 25-hydroxyvitamin D, C-Telopeptide of type I collagen (CTX-1) in 2010 and 2012. Their dietary intake was assessed in 2010 using a food frequency questionnaire, and sociodemographic and health information was collected. Four dietary patterns explained 54.4% of the variance of dietary intake, namely staple foods and processed meats, home cooking, snacking, and high sugar. Dietary magnesium negatively correlated with CTx-1 in 2012 (r = −0.21, *p* = 0.02), calcium correlated with distal radius BMD in 2010 (r = 0.22, *p* = 0.01) and 2012 (r = 0.24, *p* = 0.005), and the snacking dietary pattern score correlated with FN BMD in 2010 (r = 0.18, *p* = 0.03) and 2012 (r = 0.21, *p* = 0.02). The baseline CTx-1 and dietary magnesium intake predicted 22% of the variance in percentage change of CTx-1 over two years (*p* < 0.001).The magnesium intake predicted short-term bone resorption over two years.

## 1. Introduction

The ethnic differences in bone mineral density (BMD) and fracture risk have been described in both high-income [1,2], and low- and middle-income countries [3,4]. In South Africa, black populations have been reported to have a higher overall BMD and a lower fracture risk compared to white populations [4]. However, evidence point towards a lower than expected BMD in black South African women and an increased risk for poor bone health and fractures [5,6,7]. Reduced BMD among black South African women may be associated with the predominance of environmental exposures over genetic factors.

Earlier studies [8,9] identified that the migration of black South Africans into urban areas significantly affects their health status with an increase in the incidence of obesity and non-communicable diseases related to urbanisation, the nutrition transition, and decreased physical activity. A high body mass index (BMI) has been suggested to protect against low BMD by some [10,11], while others have found excessive fat mass to be associated with low BMD and not protecting against osteoporosis [12,13]. Similarly, lean mass and not fat mass strongly correlated with bone health in black South African women [14]. Therefore, the high prevalence of obesity among black South African women [15] may be a contributing factor to a lower BMD in this group. In addition to high fat mass levels, increased circulating inflammatory cytokines in obese individuals have also been linked to a low BMD [16].

Diet is a known modifiable risk factor for optimal bone health, with the value of specific nutrients such as dietary calcium and vitamin D well established [17]. More recently, a holistic approach acknowledging the interaction between foods, nutrients, and food components on health outcomes, including bone health, was proposed to investigate the association of dietary patterns with the markers of bone health. The dietary patterns associated with reduced bone health are characterised by the high intakes of refined cereals, processed meats, fried foods and sweet foods [18,19], while patterns high in fruit, vegetables, and a prudent diet pattern are positively associated with bone health [19,20,21]. In the Northwest Province of South Africa, an accelerated nutrition transition among black Africans resulted in a shift from a plant-based diet to an animal food-based diet which is energy-dense but nutrient poor [22] and therefore, may negatively impact bone health.

Therefore, the purpose of this prospective study was to investigate the association between nutrient intake and dietary patterns (exposures) with changes in bone turnover and BMD (bone health outcomes) in postmenopausal urban black South African women over two years.

## 2. Materials and Methods

### 2.1. Study Design

This study was part of the larger multinational Prospective Urban and Rural Epidemiology (PURE) study aimed at tracking the effects of lifestyle and changing environment exposures on the development of non-communicable diseases in populations at different stages of epidemiologic transition over ten years [23]. The South African Northwest Province (NWP) arm of the PURE (PURE-SA-NWP) study commenced with a baseline data collection in 2005 [24]. For this study, 355 black urban women were available in 2010 from the urban PURE-SA-NWP female participants, and 162 of these women were ≥ 50 years old and postmenopausal. Those who underwent measurements of distal radius BMD, dual-energy X-ray absorptiometry (DXA), and had their blood profiles done in both 2010 and 2012 (*n* = 144) were eligible for inclusion in this study. Cross-sectional dietary data, sociodemographic, and health information collected in 2010 were used to investigate changes in bone turnover and BMD over two years. All measurements for each participant were conducted on the same day. The study was approved by the Health Research Ethics Committee of the North-West University (NWU), Potchefstroom campus (NWU-00016-10-A1). All participants provided written informed consent.

### 2.2. Anthropometry, Bone Mineral Density and Bone Turnover Markers

Height was measured to the nearest 0.1 cm with a stadiometer (Leicester height measure, Seca, Birmingham, UK) and weight was determined on a portable electronic scale to the nearest 0.01 kg (Precision Health Scale, A & D Company, Japan) by anthropometrists according to the standard methods of the International Society for the Advancement of Kinanthropometry (ISAK) [23]. BMI was also calculated (weight in kilograms divided by height in meters squared).

The bone density of the distal radius was measured with a DTX-200 peripheral DXA system (Osteometer Meditech, Hawthorne, Calif., USA). The femoral neck (CV = 1.2%), hip (CV = 0.8%), and anterior–posterior spine BMD (L1–L4, Spine, CV = 0.7%) were measured by a registered radiographer with a DXA (Hologic Discovery W; Hologic Inc., Waltham, Mass, USA). Measurements from the non-dominant side of each participant were used for data analysis.

Registered nurses collected a fasting blood sample from the antecubital vein using a sterile winged infusion set and syringes. The serum samples were prepared and stored in aliquots in cryotubes at −80 °C. Serum parathyroid hormone (PTH), 25-hydroxyvitamin D (25(OH)D3), and C-Telopeptide of type I collagen (CTx-1) concentrations were measured using the Roche Elecsys 2010 COBAS system (Roche Diagnostics, Indianapolis, IN, USA). The high-sensitivity C-reactive protein (hsCRP) was measured using a particle-enhanced turbidimetric assay [24].

### 2.3. Questionnaires

Structured questionnaires were used to collect socio-demographic and lifestyle information, including medication and tobacco use [25] in 2010. Questionnaires were administered by trained fieldworkers in the language of choice of the study participants. A validated culturally sensitive quantitative food frequency questionnaire (QFFQ) [26] and a modified Baecke physical activity questionnaire validated for this population [27] were used, as previously described by Kruger and colleagues [28], to assess dietary intake and physical activity levels in 2010. A score of 1 to 3.3 representing low physical activity, 3.34 to 6.67 moderate physical activity, and a value greater than 6.67 a high level of physical activity, was used.

### 2.4. Nutrient Intake and Dietary Patterns

The food intake was coded and analysed using the South African Medical Research Council database [29]. The 100 individual food items were manually allocated into 40 food groups. Foods were grouped according to their similarity in nutrient profile or culinary usage. Food groups were then further reduced down to 13 food groups during exploratory factor analysis until a value of 0.7 was reached for the Kaiser–Meyer–Olkin (KMO) measure of sampling adequacy. The final food group list that was subsequently used to identify food patterns is listed in Table 1.

### 2.5. Oblique Rotation Principal Component Factoring

A principal component analysis was conducted on the 13 food group items with varimax rotation to achieve a simpler structure with excellent interpretability. The KMO measure of 0.776 verified the sampling adequacy for the analysis and all KMO values for the individual items were greater than 0.6, which is well above the acceptable limit of 0.5 [30]. An initial analysis was run to obtain eigenvalues for each factor in the data. Four factors had eigenvalues over Kaiser’s criterion of 1 and in combination explained 54.4% of the variance. The scree plot was ambiguous and showed inflections that would justify retaining either two or four factors. We retained four factors based on Kaiser’s criterion. Food groups with a factor loading of ≥0.5 on a component were considered informative for the interpretation of the dietary patterns. All participants were then scored on each of the dietary patterns using Bartlett’s method [31]. Further partitioning by score took place by dividing participants into tertiles. A comparison of baseline characteristics was performed across each tertile.

### 2.6. Statistical Analysis

Data were analysed with IBM SPSS version 24 (IBM Company, Armonk, NY, USA). A statistical significance level of 0.05 was used throughout. The distribution of continuous variables was assessed using Q-Q plots and the Shapiro–Wilk test for normality. BMD and 25(OH)D3 had a normal distribution, but PTH, CTx-1, and dietary variables deviated from the normal distribution. Non-parametric tests were used to compare the differences between these variables. BMD, CTx-1, PTH, and 25(OH)D3 changes were calculated as the % change from baseline.

The dependent t-test was used to test for differences between the normally distributed variables collected in 2010 and 2012, while the Wilcoxon signed-rank test was used to compare variables with a non-normal distribution. Spearman correlation analysis was performed to determine correlations between baseline variables, as well as between changes in BMD, PTH, and CTx-1 from baseline, and age, height, BMI, physical activity, CRP, dietary patterns, and nutrient intakes. The predictors of the percentage change in CTx-1 from baseline were determined using multiple regression models, with baseline CTx-1, baseline hsCRP, weight, height, age, physical activity index, dietary magnesium and calcium, alcohol intake, and tobacco use as covariates.

## 3. Results

### 3.1. Participant Characteristics, Nutrient Intake, and Dietary Patterns

Participant characteristics are described in Table 2. Most women had a waist circumference above the 80 cm recommendation [32] and had a low physical activity level [28].

The dietary calcium intake was below the estimated adequate requirements (EAR = 1000 mg/day) in 91.5% women; 21.1% had magnesium intakes below the EAR for women older than 51 years (EAR = 265 mg/day) [33].

There were significant positive correlations between the total calcium intake (r = 0.22, *p* = 0.01), as well as dairy foods (milk, milk drinks, and cheese) (r = 0.21, *p* = 0.02) and distal radius BMD at baseline (2010) and in 2012 (r = 0.24, *p* = 0.01 and r = 0.27, *p* = 0.001, respectively). Magnesium negatively correlated with CTx-1 in 2012 (r = −0.21, *p* = 0.02).

The factor analysis of dietary intakes identified four dietary patterns according to Kaiser’s criterion. These extracted factors explained 54.4% of the variance of the dietary intake variables. The first pattern (explaining 17.3% of the variance) presented with high positive loadings for starchy cooked foods, breads, ‘vetkoek’ (fried bread cakes), margarine, dairy foods (milk, cheese, and yoghurt), sugars, and processed meats and was named *staple foods and processed meats*. The second pattern explained 16.8% of the variance and was named the *home cooking* pattern. It was characterised by the high positive loadings on vegetable salad greens, meats, eggs, oil, and dressings. The third pattern (explaining 11% of the variance) was characterised by high loadings on fruit, crackers, and savoury snacks, as well as starchy vegetables including hot chips and was named the *snacking* pattern. The fourth pattern explained 9.3% of the variance and was named the *high sugar* pattern, and was characterised by high loadings on soft drinks and dairy products with added sugar such as ice cream and flavoured milk. Participants with a higher adherence to the *snacking* pattern were heavier than those with a lower adherence to the *snacking* pattern (Supplementary Materials Table S1) and had a higher femoral neck BMD (*p* < 0.05).

The calcium intake correlated positively with all four dietary patterns, namely the *snacking* pattern (r = 0.49, *p* < 0.001), the *staple foods and processed meats* pattern (r = 0.35, *p* < 0.001), the *home-cooking whole foods* pattern (r = 0.31, *p* < 0.001), and the *high sugar* pattern (r = 0.28, *p* < 0.001). The magnesium intake was positively associated with the *staple foods and processed meats* pattern (r = 0.46, *p* < 0.001) and the *home-cooking whole foods* (r = 0.28, *p* < 0.001) pattern. A weak positive correlation was found between the *snacking* pattern and femoral neck BMD at baseline (r = 0.18, *p* = 0.03) and 2012 (r = 0.21, *p* = 0.02). No other correlations were found between the dietary patterns and the markers of bone turnover.

### 3.2. Bone Mineral Density and Bone Turnover

CTx-1 and PTH levels increased significantly over the two years, indicating increased bone resorption (Table 3). Distal radius BMD and left femoral neck BMD decreased significantly over two years, while spine BMD remained stable (Table 3). Most of the women (70.4%) were vitamin D sufficient with serum levels above 30 ng/mL at baseline. Serum concentrations reduced over the two years but remained in the sufficient range for 55.7% of the women [34].

As expected, age correlated positively with PTH (r = 0.27, *p* = 0.002), and negatively with distal radius BMD (r = −0.41, *p* < 0.001), spine BMD (r = −0.19, *p* = 0.02) and femoral neck BMD (r = −0.24, *p* = 0.005) at baseline, but not with CTx-1. CTx-1 correlated negatively at baseline and at 2012 with distal radius BMD (r = −0.29, *p* < 0.0001) and spine BMD (r = −0.22, *p* = 0.008). There were positive correlations between baseline BMI and PTH (r = 0.28, *p* = 0.001), distal radius BMD (r = 0.47, *p* < 0.001), spine BMD (r = 0.40, *p* < 0.001), and femoral neck BMD (r = 0.62, *p* < 0.001). Positive correlations between baseline (2010) weight, WC, and hip circumference were also shown with all BMD sites at baseline and 2012 (*p* < 0.05).

There was a positive correlation between the change in the distal radius (r = 0.26, *p* < 0.001) and femoral neck BMD (r = 0.26, *p* < 0.001), with the change in PTH. The change in CTx-1 and the change in PTH were also significantly correlated (r = 0.31, *p* < 0.001). The change in 25(OH)D3 levels over the two years correlated negatively with the change in CTx-1 (r = −0.26, *p* = 0.003), as well as the change in PTH (r = −0.39, *p* < 0.001).

### 3.3. Regression Analyses

Stepwise multiple regression analyses revealed that CTx-1 at baseline and magnesium intake (negative association) can be used to predict the percentage change in CTx-1 over two years, as illustrated in Table 4.

## 4. Discussion

The key finding of this study was that baseline CTx-1 and dietary magnesium intake predicted 22% of the variance in percentage change in CTx-1 over two years in this group of postmenopausal urban black South African women. There were positive correlations between distal radius BMD and dairy food and calcium intake. Dietary magnesium intake was negatively associated with bone resorption. Four dietary patterns were identified, of which the *snacking* pattern had a weak positive association with femoral neck BMD. Bone resorption increased and BMD declined over two years, indicating a reduction in bone health.

The role of diet and nutrition on bone health is complex, as nutrients and food components may have beneficial or detrimental effects on BMD and/or bone turnover [35]. More recently, dietary patterns have shown associations with BMD. Dietary patterns allow for the investigation of the cumulative effect of a particular diet on health outcomes, such as bone health [36]. In a population-based prospective cohort study (*n* = 5144), the Rotterdam Study [37], a higher BMD was found in those following a *traditional* (high in potatoes, meat, and fat) and *health-conscious* dietary pattern (high in fruit, vegetables, poultry, and fish). On the other hand, a *processed* dietary pattern (high in processed meat and alcohol) was associated with lower BMD. Other studies have also shown that dietary patterns high in plant-based food, low-fat dairy, and fish positively impact bone health [20,38] and that a Western-style diet high in processed food, alcohol, high-fat dairy, and refined grains negatively impacts bone health [19,21]. We found a weak association between the *snacking* dietary pattern and femoral neck BMD. The weak correlation between the *snacking* pattern and BMD may have been mediated through body weight as women that showed a higher adherence to the *snacking* pattern were heavier and had a higher femoral neck BMD. A lack of association between dietary patterns and bone health markers in the current study may be due to the small sample size, as well as the overall low diet quality across all four dietary patterns [22].

Adequate dietary calcium plays an important role in bone mineral density acquisition in children and young adults, however the role of dietary calcium alone on bone health in postmenopausal women is less clear [39]. In the current study, dietary calcium was associated with radius BMD at baseline and follow-up, but at no other BMD sites and not with any change in bone resorption over two years. Our findings are similar to other cross-sectional studies with no relationship between dietary calcium intake and BMD at the spine and femoral neck in postmenopausal women from Saudi [40]. Our findings also support the literature indicating that calcium intake was not associated with PTH or bone resorption when serum vitamin D levels are sufficient [41].

Interestingly, low dietary magnesium intake in the current study was associated with an increase in bone resorption (CTx-1) over two years and dietary magnesium was a predictor of change in bone resorption. Similar to our findings, New et al. [42] found a negative association between bone resorption markers excretion and dietary magnesium intake in post-menopausal women. In addition, low serum magnesium levels have been implicated in low bone strength and low magnesium levels were found in women with low bone density [43]. The importance of dietary magnesium in maintaining BMD may be attributed to its role in skeletal acid-base balance [42]. Our findings on the potential role of dietary magnesium in bone health together with those of others are compelling and warrant further investigation.

In our cohort of women, higher levels of CTx-1 at baseline were significantly associated with the increased loss of BMD at the distal radius and spine over two years. These findings are in line with evidence supporting bone resorption as a predictor of future bone loss [44]. Furthermore, PTH was positively correlated with age, as well as with CTx-1 levels which increased over the two years. In addition, change in distal radius and femoral neck BMD, as well as change in CTx-1 correlated significantly with the change in PTH. These results confirm the findings of Arabi et al. [45] who reported that PTH levels, but not serum vitamin D levels, predict bone loss rates in older people. In their cohort, changes in vitamin D status did not relate to changes in BMD, while PTH was negatively correlated with changes in BMD at all skeletal sites. Similarly, others [40] found negative correlations between PTH and BMD, as well as PTH to be a predictor of BMD at the lumbar spine and femoral neck in postmenopausal Saudi women. Eastell et al. [46] reported that PTH may also respond to low serum vitamin D levels. In our study, most of the women were vitamin D sufficient, although the mean status of the women declined over the two years which was unrelated to seasonal change [47]. Therefore, the rise in PTH over time in this study may be related to age as well as the reduction in serum vitamin D levels.

Due to the cross-sectional nature of the study, causation cannot be implied in our findings and small sample size limit extrapolation to all black postmenopausal women. Despite these limitations, our findings add to the existing body of evidence on the potential role of dietary magnesium as a predictor of future bone health in a sample of black urban South African women.

## 5. Conclusions

This study aimed to shed light on the association between nutrients and dietary patterns with the change in the bone health of postmenopausal black South African women. Our results highlight the potential role of dietary magnesium as a predictor of bone resorption, the association between dietary calcium and dairy food with distal radius BMD, and identified other non-dietary factors that play a role in the bone health of this group of women, such as age, body mass index, and PTH.

## Figures and Tables

**Table 1:** The food group list used to identify dietary patterns.

Food Group	Foods in the Group	Number
**Starches and grains**	Starchy grains (cereals, pasta, rice, mealies/corn, samp), cooked porridge, and maize-based drinks	1
**Bread**	White, brown, and whole-wheat bread and rolls, ‘vetkoek’ (fried rolls)	2
**Vegetables**	All fresh and canned vegetables, excluding starchy vegetables	3
**Starchy vegetables**	Starchy vegetables (potato, sweet potato), fried hot chips	4
**Fruit**	All fresh and dried fruit, (mostly apples, pears, bananas, oranges)	5
**Dairy**	Milk and milk products (fresh and sour), yoghurt, cheese	6
**Sweetened milk products**	Custard sauce, ice-cream, milk desserts, dairy-juice drinks	7
**Unprocessed meat**	Meat, chicken, fish, and products, animal protein stew with potato and/or vegetables	8
**Processed meat**	Processed meats, e.g., frankfurters, viennas, ham, bacon, boerewors	9
**Fats**	All fats and oils (excluding ice cream), butter, margarine, oil, lard, salad dressings	10
**Sugars**	Sugar, syrups, sweets, jam	11
**Savoury and sweet baked/fried starchy foods**	Savoury snacks, dry crackers, popcorn, cakes, biscuits, cheese crisps, commercial dry potato chips	12
**Drinks**	Sugar-sweetened drinks, fruit juice	13

**Table 2:** The baseline descriptive and dietary data collected in 2010.

Variable	Total Group (*n*)	Median	IQR ^1^
**Age (y)**	144	59.4	54–66
**Weight (kg)**	144	67.4	55.5–80.2
**BMI ^2^ (kg/m^2^)**	144	27.9	22.8–33.0
**Waist circumference (cm)**	141	86.8	76.9–95.3
**Physical activity score**	141	2.96	2.62–3.12
**Energy intake (MJ/d)**	142	11.2	8.7–13.5
**Percentage protein intake (%)**	142	12.6	11.2V14.3
**Protein per body weight (g/kg)**	142	1.2	0.9–1.7
**Percentage carbohydrate intake (%)**	142	54	48–58
**Added sugar (g/d)**	142	62	34–93
**Fiber (g/d)**	142	30	21-41
**Percentage fat intake (%)**	142	26	22–31
**Calcium intake (mg/d)**	142	540.4	358–708
**Magnesium intake (mg/d)**	142	367.0	273–508
**Alcohol intake (g/d)**	142	0	0V5.1
**Variable**	***N***	***n***	**Percentage (%)**
**Waist circumference cut-off** **<80 cm** **≥80 cm** **≥120 cm**	142	42 100 -	29.6 70.4 -
**Smokers**	141	11	7.8
**HIV ^3^ positive**	144	12	8.3

^1^ IOR, Interquartile range; ^2^ BMI, Body mass index; ^3^ HIV, Human Immunodeficiency Virus.

**Table 3:** The change in bone mineral density, body mass index, and bone turnover over two years.

Variable	*N*	2010	2012	*P*-Value	d ^4^
**Distal radius BMD ^1^ (g/cm^2^)**	134	0.42 (0.1)	0.3 (0.14)	<.001	0.3
**Spine (L1-L4) BMD ^1^ (g/cm^2^)**	143	0.84 (0.15)	0.83 (0.15)	0.090	0.14
**Femoral neck of the hip BMD ^1^ (g/cm^2^)**	142	0.83 (0.14)	0.81 (0.14)	<.001	0.38
**BMI ^2^ (kg/m^2^)**	144	27.9 (22.8–33.0)	27.7 (22.8–33.3)	0.001	
**High sensitivity C-reactive protein (mg/L)**	141	4.83 (1.70–9.94)	4.42 (2.24–8.26)	0.720	
**C-Telopeptide of type 1 collagen (CTx-1) (ng/mL)**	132	0.47 (0.31–0.69)	0.54 (0.35–0.80)	0.004	
**Parathyroid hormone (ng/mL)**	132	41.4 (29.1–55.6)	45.8 (35.5–63.3)	<0.0001	
**25(OH)D3 ^3^ (ng/mL)**	132	35.6 (27.4–46.4)	30.7 (23.1–36.8)	<0.0001	

Parametric variables reported as mean with standard deviation, non-parametric variables reported as median, and Interquartile ranges (25th–75th percentile). ^1^ BMD, Bone mineral density; ^2^ BMI, Body mass index; ^3^ 25-hydroxyvitamin D. *P*-value indicates a significant difference between 2010 and 2012 values; ^4^ effect size of the difference between 2010 and 2012 values.

**Table 4:** Multiple regression analyses for percentage change in C-Telopeptide of type 1 collagen as dependent variable.

	Standardised β	*P*-Value
Final Model *		
**CTx-1 ^1^ Baseline (ng/mL)**	−0.45	<0.0001
**Magnesium Intake (mg)**	−0.175	0.03
**HIV infected**	0.131	0.096
**Adjusted R-squared**	0.220	

* Final model adjusted for age, height, weight, CRP, physical activity, HIV status, tobacco use, alcohol intake, and calcium intake; ^1^ CTx-1, C-Telopeptide of type 1 collagen.

## Supplementary Materials

The following are available online at https://www.mdpi.com/2072-6643/11/10/2519/s1, Table S1: Baseline characteristics of participants per tertile of adherence to the “staple food and processed meats”, “home-cooking and whole foods”, “snacking”, or “high sugar” dietary patterns.
