# Supplementary material for: Magnesium Intake Predicts Bone Turnover in Postmenopausal Black South African Women
Source: Nutrients. 2019 Oct 18;11(10):2519. doi: 10.3390/nu11102519 (PMC6836205; doi:10.3390/nu11102519)
Supplement: Supplementary file 1 [file nutrients-11-02519-s001.pdf]

Supplementary Table 1: Baseline characteristics of participants per tertile of adherence to the "staple food and processed meats", "home-cooking and whole foods", "snacking", or "high sugar" dietary patterns

	"Staple food and processed meats" pattern			"Home-cooking and whole foods" pattern			"Snacking" pattern			"High sugar" pattern		
	1st tertile	2nd tertile	3rd tertile	1st tertile	2nd tertile	3rd tertile	1st tertile	2nd tertile	3rd tertile	1st tertile	2nd tertile	3rd tertile
Age (yr)	61±8	63±10	59±7.5	61±10	61±9	61±7	61±9	61±9	61±10	61±8	61±10	60±8
Body mass index (kg/m^2)	28±8	29±7	28±7	29±7	28±7	28±7	26±6[‡]	28±6[‡]	31±8	29±7	27±7	29±7
Weight (kg)	69±19	68±19	69±16	69±18	69±19	68±18	63±17[†,‡]	67±14	75±20	69±18	66±18	70±19
Waist circumference (cm)	86±13	87±15	85±12	86±14	86±12	86±13	80±14[†,‡]	87±10	91±13	88±12	83±14	88±13
CTx-1[1] (ng/ml)	0.54±0.3	0.49±0.2	0.54±0.3	0.48±0.2	0.58±0.3	0.51±0.3	0.50±0.23	0.57±0.32	0.49±0.3	0.53±0.3	0.56±0.3	0.48±0.3
PTH[2] (ng/L)	43±21	51±27	45±28	45±17	48±31	46±27	46±24	52±33	41±17	42±19	51±29	45±27
25(OH)D3[3] (ng/ml)	37±12	36±13	37±14	37±12	35±13	38±13	39±15	35±13	36±10	37±13	34±14	38±12
Distal radius BMD[4] (g/cm^2)	0.41±0.1	0.40±0.1	0.44±0.1	0.43±0.1	0.40±0.1	0.42±0.1	0.41±0.1	0.41±0.1	0.43±0.1	0.42±0.1	0.40±0.1	0.43±0.1
Spine BMD (g/cm^2)	0.84±0.2	0.83±0.2	0.85±0.1	0.85±0.1	0.84±0.2	0.83±0.1	0.84±0.2	0.84±0.2	0.83±0.2	0.84±0.2	0.82±0.2	0.86±0.2
Femoral neck BMD (g/cm^2)	0.84±0.1	0.80±0.1	0.84±0.2	0.83±0.2	0.82±0.2	0.83±0.1	0.79±0.1[‡]	0.83±0.1	0.84±0.2	0.83±0.1	0.81±0.2	0.84±0.2
Physical activity Index	7.1±1.2	7.7±1.2	7.4±1.4	7.3±1.1	7.7±1.3	7.2±1.4	7.6±1.4	7.1±1.1	7.5±1.3	7.5±1.2	7.2±1.1	7.4±1.5

Data reported as mean with standard deviation. [1]C-Telopeptide of type 1 collagen; [2]Parathyroid hormone; [3]25-hydroxyvitamin D; [4]Bone mineral density. [‡]Significantly different from "snacking" pattern tertile 3 ($p < 0.05$); [†]Significantly different from "snacking" pattern tertile 2 ($p < 0.05$).
